# GO-DEVS: Storage and Retrieval System for DEVS Models Using Graph and Ontology Representation

**DOI:** 10.3390/s21206771

**Published:** 2021-10-12

**Authors:** Chun-Hee Lee, Jang Won Bae, Euihyun Paik

**Affiliations:** 1 Intelligence Information Research Division, Electronics and Telecommunications Research Institute, Daejeon 34129, Korea; ch.lee@etri.re.kr (C.-H.L.); ehpaik@etri.re.kr (E.P.); 2 School of Industrial Management, Korea University of Technology and Education, Cheonan 31253, Korea

**Keywords:** DEVS, storage/retrieval system, model sharing, ontology, graph

## Abstract

DEVS is a powerful formal language to describe discrete event systems in modeling and simulation areas and useful for component-based design. One of the advantages of component-based design is reusability. To reuse or share DEVS models developed by many other modelers, a system to systematically store and retrieve many DEVS models should be supported. However, to the best of our knowledge, there does not exist such a system. In this paper, we propose GO-DEVS (**G**raph/**O**ntology-represented **DEVS** storage and retrieval system) to store and retrieve DEVS models using graph and ontology representation. For effective model sharing, an ontology is introduced when a DEVS model is developed. To search for DEVS models in an effective and efficient way, we propose two types of queries, *IO query and structure query*, and provide a method to store and query DEVS models on an RDBMS. Finally, we experimentally show GO-DEVS can process the queries efficiently.

## 1. Introduction

DEVS (Discrete EVent System Specification), which was proposed by Zeigler et al., is a formal language to describe discrete event models [1,2,3]. DEVS has been used widely in modeling and simulation areas [4,5,6] because it has a well-formed formalism and models can be represented by modular and hierarchical concepts in the DEVS formalism [1,2,3]. If we build discrete event models using DEVS, the models can have comprehensive representations by its formal descriptions and can be used as building blocks for the future models by its modular property. The basic building block of DEVS is an atomic model which is similar to a finite state machine. A state is changed to the other state by an incoming message or time out. Notice that we use message, event and port interchangeably in this paper. A coupled model is composed by assembling atomic or coupled models through couplings. Since a subpart of the coupled model is a DEVS model, we can construct complex models using the hierarchical specification of DEVS. Because of the above characteristics of DEVS, it is very useful for component-based design. One of the advantages of component-based design is reusability. Using the component-based design concept, a complex system can be easily built by assembling components, which can be implemented directly by system developers themselves or borrowed from subparts of other systems for reuse. To reuse or share DEVS models developed by many other modelers, a system to store and retrieve a large number of DEVS models effectively and efficiently should be supported. However, to the best of our knowledge, there does not exist such a system. Therefore, we propose GO-DEVS (**G**raph/**O**ntology-represented **DEVS** storage and retrieval system) to systematically store and retrieve a huge number of DEVS models using graph and ontology representation.

For effective DEVS model sharing among model developers, an ontology is introduced in GO-DEVS. Ontology is a conceptualization to represent knowledge with objects and their relationships [7]. Model developers should use terminologies in ontology when they specify input and output events. To search for DEVS models from a DEVS model database, GO-DEVS supports two types of queries, *IO query* and *structure query*. The IO query is to find compatible components with the given input/output specification while the structure query is to find similar components to the given component structure. To process those queries, GO-DEVS transforms a DEVS model into the set of graphs with meta information by decomposing the model hierarchically. The transformed data are stored on an RDBMS (Relational DataBase Management System). Additionally, by adopting the XML encoding scheme, GO-DEVS improves the retrieval efficiency significantly. The contributions of the paper are as follows:

- **Introduction of an Ontology to DEVS Models:** To effectively reuse DEVS models developed by other developers, models should be understood in common. To do that, we introduce an ontology to DEVS models.
- **Transformation of a DEVS Model into Graph Representation:** To support the efficient model retrieval, we need to represent DEVS models effectively because they show a complicated pattern and are not easy to handle. To relieve the complexity of DEVS model structures, we propose a method to represent them by the set of graphs with meta information.
- **Queries to Retrieve DEVS Models:** To retrieve DEVS models that model developers want to find, we propose two types of queries, *IO query* and *structure query*.
- **Effective Table Design to Retrieve DEVS models:** To support efficient processing for two types of queries, we provide the basic table design and the advanced table design. In the advanced table design, an XML numbering scheme is adopted.

## 2. Related Work

Originally, Zeigler et al. invented DEVS formalism [1,2]. After that, many researchers have been extending DEVS [8,9]. Dalle and Zeigler formally embedded a component sharing concept into DEVS, where multiple instances of a DEVS component can share identical internal states [8]. Vicino et al. proposed a new time data type in DEVS [9]. Additionally, simulators to execute DEVS models have been developed in various languages such as C++, Java, and Python [10,11,12].

SES (System Entity Structure) was proposed to represent the structural knowledge in hierarchical and modular systems and provide “plan-generate-evaluate” framework [2,13]. In terms of model reusability, its aim is similar to GO-DEVS. However, SES does not consider the relations between components at the same level, which show a graph pattern.

Additionally, Song et al. [14] provided a conceptual methodology for model-verification in Model-Driven Architecture (MDA) using simulation models. To map models (described by ontologies) in MDA with models (described by SESs) in simulations, Song et al. used the ontology matching algorithm. However, they tried to solve the problem of verifying models in MDA while we tried to focus on storage and retrieval systems.

In the literature of databases, indexing various types of data for fast retrieval has been studied for a long time. In particular, XML and graph data look similar to DEVS models. To process large scale XML data, XML indexing and query processing techniques have been developed [15,16]. To index and retrieve graph data, much work has been conducted [17,18,19]. Even though many XML indexing and graph indexing techniques have been invented, they cannot be applied to DEVS model storage and retrieval systems for many reasons. First, since DEVS model structures are constructed layer by layer and their internal structure shows a graph pattern, they cannot be represented by trees. Additionally, it is not easy to effectively represent them by a simple form of graphs. Second, the query forms for XML and graph data are not intended for retrieving data in the form of DEVS models. Third, the approaches in XML and graph areas do not consider model sharing among users.

In addition, query optimization has been studied extensively in the database field to optimize the processing of queries in terms of execution times. In particular, in relational databases, a query optimizer is used for finding the best query execution plan [20]. It makes multiple candidate query evaluation plans and chooses the best plan using various techniques, such as indices, query result size estimation, early selection operation processing, query rewriting with materialized views [20,21]. In [21], query optimization using materialized views was surveyed in detail.

In many cases, studies in the modeling and simulation (M&S) area have undervalued the computation cost or ignored the issues of storing and retrieving data. They are interesting problems for researchers in the database (DB) area. Therefore, in this paper, we try to bridge two areas in terms of DEVS model sharing.

## 3. Preliminary

The atomic model of DEVS (**D**iscrete **EV**ent **S**ystem Specification) is defined below [2,3].

**Definition** **1.** *An atomic DEVS model AM is formally defined by*

*AM=$<X,Y,S,s_{0},\delta_{ext},\delta_{int},\lambda ,ta>$, where*

$$\begin{array}{c} \begin{array}{ccc} & X\;is\;the\;set\;of\;input\;events, & \\ & Y\;is\;the\;set\;of\;output\;events, & \\ & S\;is\;the\;set\;of\;model\;states, & \\ & s_{0}\in S\;is\;the\;initial\;model\;state, & \\ & \delta_{ext}:Q\times X\to S,an\;external\;transition\;function,\;\;\;\;\;\;\;\;\;\;\;\;\;\;\;\;\\ & \;\;\;\;(Q=\{(s,e)|s\in S,\;0\leq e\leq ta(s)\}) & \\ & \delta_{int}:S\to S\;\;an\;internal\;transition\;function, & \\ & \lambda :S\to Y,an\;output\;function, & \\ & ta:S\to R^{+},a\;time\;advance\;function & \end{array} \end{array}$$

**Figure 1:**
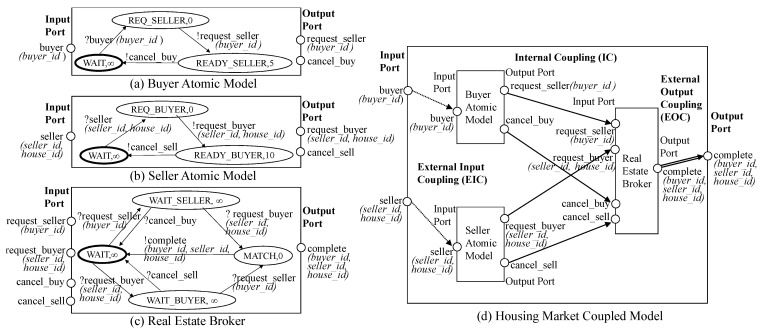
Atomic and Coupled DEVS Model Examples for Housing Market.

Basically, an atomic DEVS model can be represented by a state diagram which is similar to a finite state machine (See Figure 1a–c). The atomic DEVS model starts from the initial state $s_{0}$. After receiving an input event (∈X), the current state is changed to the other state. The state change is described by the external transition function $\delta_{ext}:Q\times X\to S$ and is bounded by the time advance function ta. The atomic DEVS model has a different property from the finite state machine. It has transitions by time out, which are described by the internal transition function $\delta_{int}$. Specifically, each state has the time limit specified by ta and if there is no incoming event during the time limit, the state is automatically moved to the other state by $\delta_{int}$. Additionally, right before calling the internal transition, the output function λ is called and generates some output events.

Figure 1a–c are atomic model examples for housing market modeling, which can be described by a state diagram. An input port and an output port correspond to one element of *X* and *Y*, respectively. The circle in the state diagram has two elements, state *S* and time advance ta. The thick circle is the initial state $s_{0}$. The arrows mean transitions by $\delta_{ext}$ or $\delta_{int}$. The arrows with the message starting with ? represent $\delta_{ext}$ while the arrows with the message starting with ! represent $\delta_{int}$ and λ. Initially, the buyer model (Figure 1a) waits to receive the buyer input event. We set the time advance of the initial state WAIT to +∞ to change the state by only external transitions. After receiving the input event, the current state of the model is changed from WAIT to REQ_SELLER. As soon as we arrive at the REQ_SELLER state, the buyer model generates the output event *request_seller* and the current state is changed to READY_SELLER since REQ_SELLER has 0 time advance. The *request_seller* event will be sent to the real estate broker model (Figure 1c) and the state of the model will be changed from WAIT to WAIT_SELLER. By the state diagrams, we can understand how the atomic models work.

A coupled DEVS model consists of subcomponents and connections (i.e., couplings) between subcomponents. Each subcomponent of the coupled DEVS model is an atomic DEVS model or a coupled DEVS model.

**Definition** **2.** *A coupled DEVS model CM [2,3] is formally defined by*

*CM=<X,Y,M,EIC,EOC,IC,Select>, where*

$$\begin{array}{c} \begin{array}{ccc} & X\;is\;the\;set\;of\;input\;events, & \\ & Y\;\;is\;the\;set\;of\;output\;events, & \\ & M\;is\;the\;set\;of\;component\;models, & \\ & EIC\subseteq CM.X\times \underset{m\in M}{⋃}m.X:external\;input\;coupling\;relations, & \\ & EOC\subseteq \underset{m\in M}{⋃}m.Y\times CM.Y:external\;output\;coupling\;relations, & \\ & IC\subseteq \underset{m\in M}{⋃}m.Y\times \underset{n\in M}{⋃}n.X:internal\;coupling\;relations, & \\ & Select:tie-breaking\;function \end{array} \end{array}$$

For each subcomponent $M_{i}$ to work together, events (i.e., event messages) should be transmitted to each other. For event transmission, DEVS introduces an input port to receive the input event and an output port to send the output event. A port is allowed to receive or send only one type of event. When the input port (resp., output port) of the root model is connected to the input port (resp., output port) of the subcomponent, it is called *EIC* (resp., *EOC*). Additionally, it is called *IC* when the output port of some subcomponent is connected to the input port of the other component. Please note that EIC, EOC and IC stand for External Input Coupling, External Output Coupling and Internal Coupling, respectively. Through recursive coupled model definition and couplings, a DEVS model is hierarchically constructed, and subcomponents of the model are connected. We omit the set of subcomponent names, *D*, in this definition. For more detailed explanation in DEVS, we can refer to [2,3]. Figure 1d shows the coupled DEVS example using the atomic models. EIC, IC and EOC are depicted by dotted lines, solid lines, and double lines, respectively.

## 4. Scenario and Architecture of GO-DEVS

**Figure 2:**
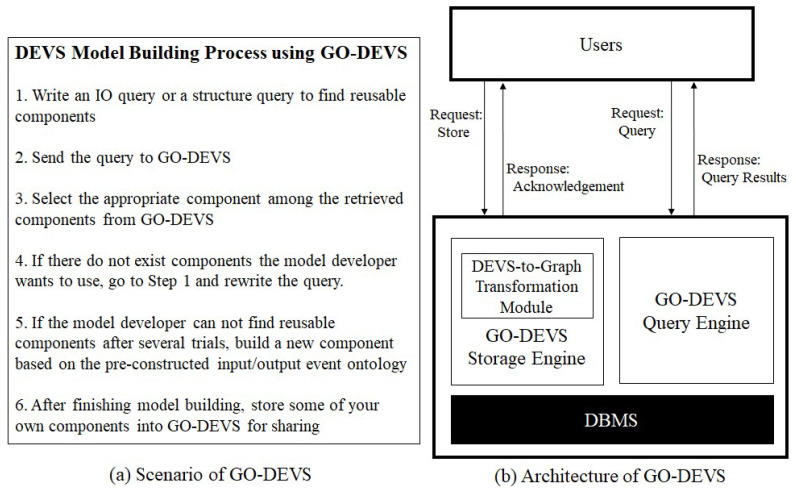
Scenario and Architecture of GO-DEVS.

GO-DEVS can be used for DEVS component sharing. Figure 2a shows the scenario how a DEVS model developer can use GO-DEVS when the model developer builds a model. Before DEVS model construction, the model developer searches for reusable components using GO-DEVS. To find reusable components, the model developer should write an IO query or a structure query. After finishing writing the query, the model developer sends the query to GO-DEVS. The model developer then chooses the appropriate component among the retrieved components from GO-DEVS. However, the model developer might think that all the retrieved DEVS components are useless. In that case, there are two options for the model developer. The first option is to rewrite the query and check other retrieved components. The second option is to directly build a new component. When the model developer builds a new component, input and output ports of the component should be designed with the pre-constructed input/output event ontology. Through the processes above, the model developer can build his/her own DEVS model. After that, the model developer can store some of components into GO-DEVS for DEVS model sharing.

The architecture of GO-DEVS is illustrated in Figure 2b. GO-DEVS consists of Storage Engine and Query Engine. The storage engine transforms any DEVS model into the set of graphs with meta information and effectively stores them into a DBMS. Because DEVS models show complicated patterns and hierarchically constructed structures, we represent their structures by the set of graphs with meta information, which are easy to understand and manage. Therefore, the storage engine has a module, called DEVS-to-Graph Transformation Module, to transform a DEVS model into the set of graphs with meta information. After transforming the DEVS model, the storage engine stores it into a DBMS.

The query engine supports two types of queries, IO query and structure query. The IO query is to find reusable components by input/output event specifications while the structure query is to find reusable components by structural information. In the case of the structure query, it is not intuitive to input the structural information by text string. Therefore, the query engine might provide GUI (Graphical User Interface) to input the structure query. However, we will not deal with GUI in this paper since it is beyond the scope of this paper.

## 5. Ontology and Graph Representation in GO-DEVS

**Figure 3:**
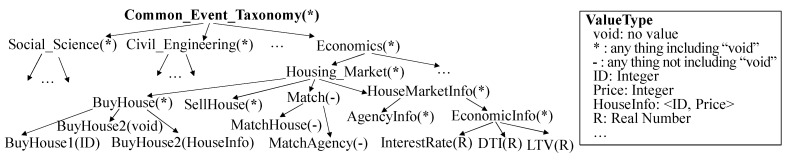
Common Taxonomy Example.

For model sharing among different model developers, a common representation language is necessary. Ontology is one of the best tools for that. Therefore, we assume that input and output events of DEVS models are specified by ontology. Even if we can use various types of well-expressive ontology representations, we use a taxonomy, one of the simplest ontology representations. This is because we do not focus on ontology representation, but on the model retrieval system. For DEVS model sharing, model developers should specify input and output events by the common event taxonomy. Figure 3 shows the example of the event taxonomy. An event in DEVS modeling might have some values to describe the detail of the event. Thus, each node in the event taxonomy is represented by *EventName(ValueType)*. *EventName* is the event name and *ValueType* is the value type of the event. When we define a value type, wild cards (i.e., void, *, -) are allowed. Please note that if event $e_{1}$ is an ancestor of event $e_{2}$ on the taxonomy, the range of the value type of $e_{1}$ should include that of $e_{2}$. Based on the event taxonomy above, an event is defined as a series of terms from the root node of the taxonomy to the target node of the taxonomy. For instance, we can describe the *BuyHouse1* event by Common_Event_Taxonomy.Economics.Housing_Market.BuyHouse.BuyHouse1.

**Figure 4:**
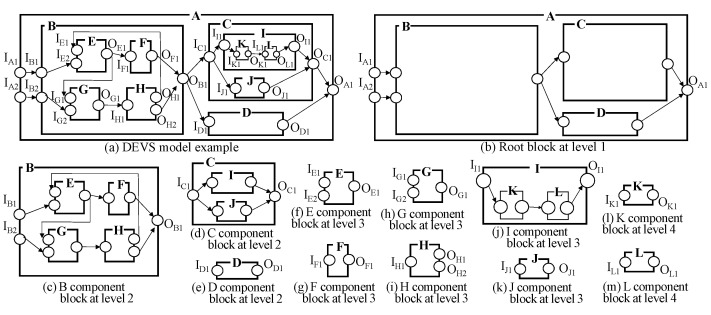
DEVS Example.

**Figure 5:**
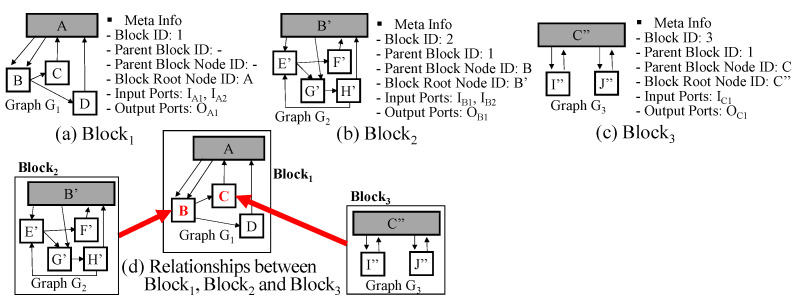
Block Units in Example of Figure 4.

A DEVS model can have a very complicated structure such as Figure 4a and is not easy to manipulate and understand. To transform the DEVS model into a simpler form, let us consider the case of a coupled model first. We can observe a coupled DEVS model layer by layer. For example, see the DEVS model of Figure 4a. Even though it has multiple layers and shows the complicated structure, we can see the top layer (i.e., level 1) first. The top layer consists of just B, C and D subcomponents as shown in Figure 4b. It has a much simpler shape than the original full structure. Then, we can observe the B component at the next layer (i.e., level 2) as shown in Figure 4c. In this way, we can observe the complicated structure of the coupled DEVS model by hiding the details of its subcomponents as shown in Figure 4b–m (Level 1: A, Level 2: B, C, D, Level 3: E, F, G, H, I, J, Level 4: K, L). For effective representation of the model structure, we introduce the block unit. Informally speaking, the block unit is the component structure without the details of its subcomponents. The block unit is illustrated in Figure 5.

The block unit has two elements <G, M>, where G is the graph and M is the block meta information. M consists of block id, parent block id, parent block node id, block root node id, input ports and output ports. The block id is the block identifier. The block is connected to some node of its parent block, which is denoted by parent block id and parent block node id. For example, Block 2 is connected to the B node of Block 1 in Figure 5d. The block root node means the component itself. Graph G represents the relationships between subcomponents or the relationships between the block root node and subcomponents. The arrows from the block root node to non-root nodes correspond to EICs and the arrows from non-root nodes to the block root node correspond to EOCs and the arrows among non-root nodes correspond to ICs. Because two nodes can be connected with several ports, there might be multiple edges between nodes.

Next, an atomic model can be represented by a graph in a different way. The atomic model can be described by a state-transition diagram and has a similar structure to a graph. See the atomic DEVS examples of Figure 1. If we consider states of the atomic model by nodes and transitions of the atomic model by edges, we can easily represent the atomic model by the graph. An atomic model is also denoted by the block unit <G,M>. However, G is not the graph for the component structure but the graph for state transitions above and the edge of G is represented in a different way. Additionally, the block root node id of M is empty. In summary, we can represent a DEVS model by multiple block units and each block unit has graph G and meta information M.

## 6. Queries for DEVS Model Retrieval

**Figure 6:**
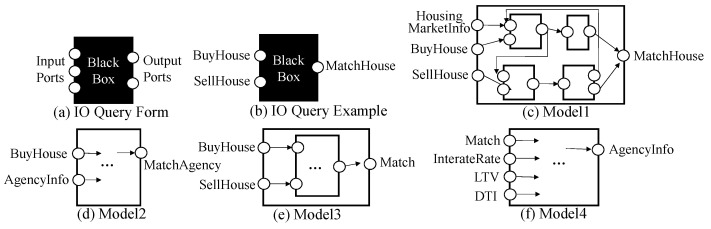
IO Query Examples.

We propose two types of queries, *IO query* and *structure query*, for effective retrieval and reuse. The IO query is a simple query to find components with the compatible input and output events. The IO query form is illustrated in Figure 6a. The query is considered to be a black box DEVS model where only input and output ports (i.e., events) are specified. Based on the event taxonomy, we can formally define the IO-compatibility among components.

**Definition** **3.** *Component A with input event set $X_{A}$ and output event set $Y_{A}$, is IO-compatible with component B with input event set $X_{B}$ and output event set $Y_{B}$, denoted by $A\to_{io}B$, if*

(1) *$\forall x_{A}\in X_{A}$, $\exists x_{B}\in X_{B}$ where $x_{B}$ is located at the same position as $x_{A}$ or the ancestor position of $x_{A}$ on the event taxonomy.*
(2) *$\forall y_{A}\in Y_{A}$, $\exists y_{B}\in Y_{B}$ where $y_{B}$ is located at the same position as $y_{A}$ or the ancestor position of $y_{A}$ on the event taxonomy.*

The IO query is to find a set of components, $C=\{c\in MDB|q\to_{io}c\}$, where q is the given IO query and MDB is a DEVS model database collected in the central server for model sharing. Figure 6 shows the IO query example. Given the IO query q in Figure 6b, GO-DEVS finds DEVS models c such that q $\to_{io}$ c. The result of the query is (c) and (e) in Figure 6. Please note that Match is an ancestor of MatchHouse (See Figure 3).

The structure query is a query to find structurally similar components. In the structure query, we focus on how subcomponents are hierarchically constructed and how subcomponents are connected. We do not handle the internal structures of atomic subcomponents because it is too detailed for users to specify. As we mentioned in Section 5, a DEVS model structure is represented by the set of block units. To find components with similar structures, we specify the pattern in a block unit and the relationship between blocks. It is called the *block statement*. Specifically, the block statement has Block Statement ID, Parent Block Statement ID, Parent Block Statement Node ID, Topological Relationship, Graph Pattern, and Root Node. **Block Statement ID** is the identifier for the block statement. It is used to refer to the relationship between blocks. One block statement corresponds to one block unit. **Parent Block Statement ID** is the identifier for the parent block statement. **Parent Block Statement Node ID** is the node identifier in the parent block statement. A block is connected to some node *V* of the parent block as explained in Section 5. Parent Block Statement Node ID corresponds to the node *V*. **Topological Relationship** has two types, (*Direct/Indirect*). The direct relationship (/) represents the parent-child relationship and the indirect relationship (//) represents the ancestor-descendant relationship. In the indirect relationship, there can exist another block between Block A and Block B, where Block A is the block corresponding to Block Statement ID and Block B is the block corresponding to Parent Block Statement ID. **Graph Pattern** is a structure specification described by graph representation. **Root Node** is the root node of the Graph Pattern. The root node means the outmost model.

**Figure 7:**
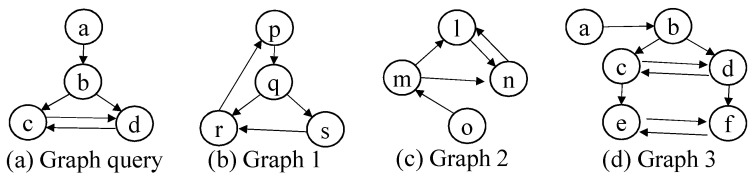
Subgraph Query Example.

For structural similarity in a block, we adopt graph pattern queries (For structural search, we consider not multigraphs but simple graphs. Even though each block is represented by a multigraph G(V,E), we can easily reduce the multigraph to the simple graph.). There are many types of graph pattern queries such as subgraph query (with/without labels), supergraph query, similar query [17,18,19,22,23,24,25]. In this paper, we use the subgraph query without labels since there is no standard graph similarity measure and the subgraph query without labels includes at least the pattern structure which the user wants to find. Figure 7 shows the subgraph query (without labels) example. Given the graph query in Figure 7a, the subgraph query finds graphs that contain the graph structure of the query without considering labels. In case (c), if we use the mapping a–o, b–m, c–n and d–l, we can say that graph (c) contains the query in terms of subgraph isomorphism. In a similar way, we are sure that (d) contains (a). Therefore, the result for the subgraph query (a) is (c) and (d).

**Figure 8:**
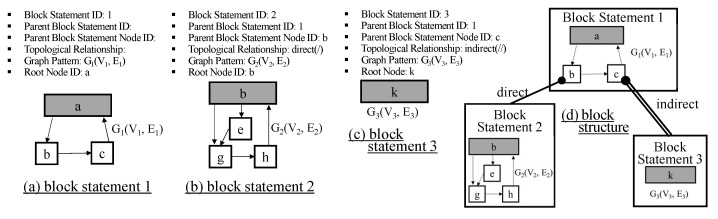
Structure Query Example.

Until now, we explained a single block statement. The structure query consists of multiple block statements. Each block B1 can be connected to another block B2, which is described by Parent Block Statement ID, Parent Block Statement Node ID, and Topological Relationship. If Topological Relationship is specified by *direct*, block B1 should be connected directly to its parent block B2, i.e., the level difference between B1 and B2 is 1. However, if Topological Relationship is specified by *indirect*, the level difference between B1 and B2 can be more than or equal to 1. Thus, the structure query finds DEVS models whose block structures meet the given multiple block statements. Figure 8 shows the structure query example with 3 block statements. Block statement 1 specifies the outmost structure. The graph pattern of block statement 1 is shown in the bottom of Figure 8a. According to block statement 2 (See Figure 8b), block 2 should be directly connected to block 1 through node b, where block 1 and block 2 correspond to block statement 1 and block statement 2, respectively. Additionally, according to block statement 3 (See Figure 8c), block 3 should be indirectly connected to node c of block 1, where block 3 corresponds to block statement 3. Relationships among 3 block statements are depicted in Figure 8d. Please note that the DEVS model in Figure 4a meets this structure query.

## 7. Storing DEVS Models in GO-DEVS

**Figure 9:**
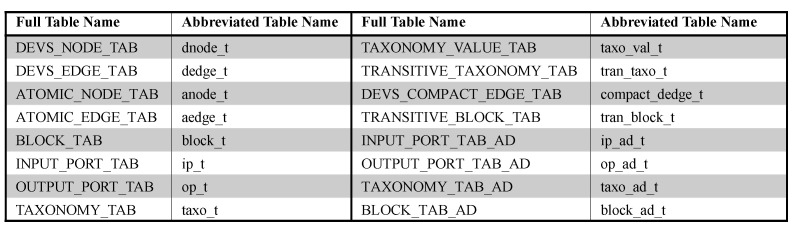
Abbreviated Table Name.

To store DEVS models, we use an RDBMS because it is very mature and stable. We will first show the basic relational table design in Section 7.1 and the advanced relational table design in Section 7.2 and Section 7.3. For convenience, we use the abbreviated table names instead of full table names as shown in Figure 9.

### 7.1. Basic Table Design

**Figure 10:**
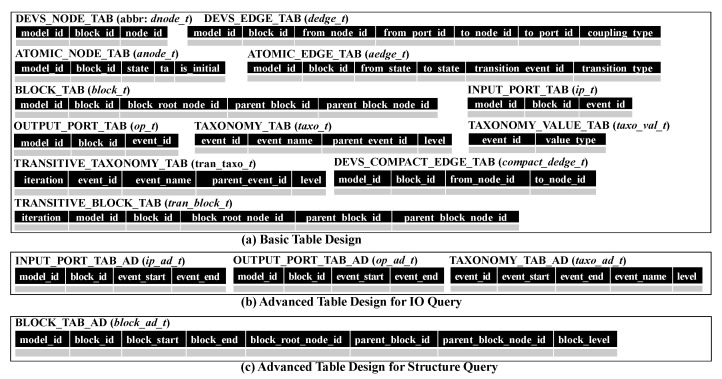
Table Designs.

In Section 5, we provided a method to transform a DEVS model into multiple blocks. A block consists of *G* and *M*, where *G* is a multigraph with nodes *V* and edges *E* and *M* is meta information. We design *dnode_t*, *dedge_t*, *anode_t* and *aedge_t* to store *G* and design *block_t*, *ip_t* and *op_t* for the meta information *M*. *dnode_t* and *dedge_t* are tables to store graph data *G* from coupled models while *anode_t* and *aedge_t* are tables to store graph data *G* from atomic models. Since graphs from coupled and atomic models show different forms, we store them to separate tables. Figure 10a shows the basic table design. *block_t* is the table to store block id, parent block id, parent block node id and block root node id in the meta information. *taxo_t* and *taxo_val_t* are the tables to store tree nodes and their values in the common taxonomy, respectively. We add the column parent_event_id to *taxo_t* to express the parent-child relationship of the taxonomy.

To process the IO query *q*, we must check if the ports of *q* and component *c* have the same path on the taxonomy tree. To do that, we should perform multiple self-joins on *taxo_t* and it will raise the heavy processing cost. Therefore, we precompute all the pairs for ancestor nodes and descendant nodes by evaluating the transitive closure of edges <event_id,parent_event_id>. The result is stored on *tran_taxo_t*.

The structure query is a query to find structurally similar components and consists of block statements. Structural characteristics can be specified by the graph pattern of the block statement. Since we use a simple graph to investigate structural similarity, we reduce multigraphs into simple graphs. We make the edges of simple graphs by running the SQL statement of "INSERT INTO *compact_dedge_t* SELECT distinct model_id, block_id, from_node_id, to_node_id FROM *dedge_t*". It is stored on *compact_dedge_t*. A block statement has the topological relation as a condition. If the topological relation is indirect, we must check if two blocks have the ancestor-descendant relationship. However, *block_t* has just the parent-child relationship. In a similar way to *taxo_t*, we can evaluate the transitive closure of *block_t* in terms of <block_id,parent_block_id>. The transitive closure is stored on *tran_block_t*.

### 7.2. Advanced Table Design for IO Query

In the basic table design, we made *tran_taxo_t* to avoid the self-join operations on *taxo_t*. However, in the case of deep taxonomy trees, much more records can be produced compared to the original table *taxo_t* and it will raise another cost to store records and join with other tables. To solve it, we can adopt the region numbering scheme [26] which is one of XML numbering techniques to check node relationships. We will briefly introduce the region numbering.

**Theorem** **1.** *[26] Suppose a tree T(V,E). We assign two numbers (* ***start*** *,* ***end*** *) to each node in V as follows. We traverse all the nodes of T in the depth-first order with* ***counter*** *. When we visit some node for the first time, we assign the* ***counter*** *value to* ***start*** *of that node. When we visit some node for the last time, we assign the* ***counter*** *value to* ***end*** *of that.* ***counter*** *increases whenever the value is assigned. Then, for any u,v ∈V, u is an ancestor of v in the tree T iff u.start < v.start and v.end < u.end.*

**Figure 11:**
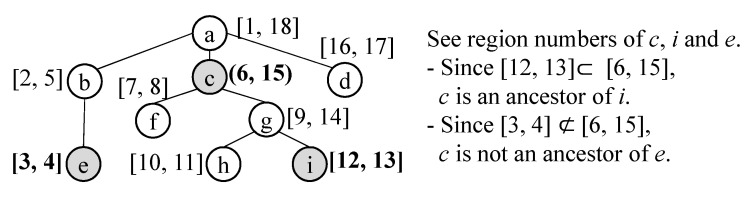
Region Numbering Example.

The conditions of u.start < v.start and v.end < u.end can be expressed by intervals, [v.start, v.end] ⊂ [u.start, u.end]. The example of the region numbering scheme is shown in Figure 11. We assign two numbers *[start, end]* to each event of the taxonomy by traversing the taxonomy tree. By the property of the region numbering scheme, we can easily check if any two events are in the ancestor-descendant relationship. Suppose that event $ev_{1}$ has $\left[s_{1},e_{1}\right]$ and event $ev_{2}$ has $\left[s_{2},e_{2}\right]$ according to the region numbering scheme. If $\left[s_{1},e_{1}\right]\subset \left[s_{2},e_{2}\right]$, we can say that $ev_{2}$ is the ancestor of $ev_{1}$. Otherwise, $ev_{1}$ and $ev_{2}$ are not in the ancestor-descendant relationship. Please note that the ancestor-descendant relationship includes the parent-child relationship and we can also know that $ev_{1}$ and $ev_{2}$ are in the parent-child relationships by checking two formulas, (1) $\left[s_{1},e_{1}\right]\subset \left[s_{2},e_{2}\right]$ and (2) $ev_{2}.level=ev_{1}.level+1$.

Based on this concept, we provide the advanced table design for input and output events in Figure 10b. In the tables for input and output ports, we use event_start and event_end instead of event_id to easily check if two events have the ancestor-descendant relationship. The tables are named *ip_ad_t* and *op_ad_t*. Additionally, in the table for the taxonomy, we add event_start and event_end. This is named *taxo_ad_t*. In the advanced table design for IO queries, we use *ip_ad_t*, *op_ad_t* and *taxo_ad_t* instead of *ip_t*, *op_t* and *tran_taxo_t*.

### 7.3. Advanced Table Design for Structure Query

The structure query contains the condition for the topological relationship between blocks. To efficiently check relationships between two blocks, we adopt the region numbering scheme in the same way as Section 7.2. A DEVS model is transformed into blocks with meta information and blocks can be constructed into the tree structure. We assign two region numbers to each block while traversing the tree structure of blocks by depth-first search. In the advanced table design, two numbers of each block are added to *block_t*. We name it *block_ad_t* as shown in Figure 10c. block_start, block_end and block_level of *block_ad_t* correspond to two region numbers of the block and the level of the block in the tree structure. Because the advanced table design for structure queries uses the region numbering scheme when we check block relationships, *tran_block_t* is not necessary.

## 8. Processing Queries in GO-DEVS

To process two types of queries, we propose a method to translate them into SQL queries. We will describe the translation of IO queries in Section 8.1 and the translation of structure queries in Section 8.2.

### 8.1. IO Query Processing

**Figure 12:**
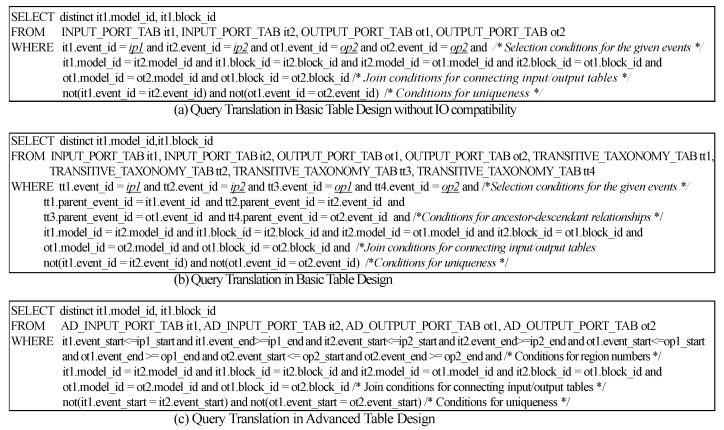
Query Translation for IO Query.

In this section, we provide a method to translate an IO query into the SQL query in the basic table design and in the advanced table design. Given IO query q=(IPs,OPs) where IPs are the set of input ports (=$\left\{ip_{1},ip_{2},\cdots ,ip_{m}\right\}$) and OPs are the set of output ports (=$\left\{op_{1},op_{2},\cdots op_{n}\right\}$), the IO query should return components *c* such that the given IOs and OPs are compatible with c. If we do not consider the IO-compatibility, we can transform the IO query into the SQL in Figure 12a. We assume $ip_{1},ip_{2},\cdots ,ip_{m}$ and $op_{1},op_{2},\cdots op_{n}$ are port IDs. By joining *m* **ip_t** and *n* **op_t** and adding selection conditions for the given input/output event IDs and conditions for uniqueness, we can write the SQL query. For simplicity, we show the query translation when *m* is 2 and *n* is 2 as an example (See Figure 12a). However, the SQL query in Figure 12a cannot find all the IO-compatible components but the components with the same ports as the given query.

To find all the IO-compatible components in the basic table design, we should use *tran_taxo_t*. *tran_taxo_t* contains all the pairs for events and their ancestor events on the taxonomy tree. For the IO query translation with the IO-compatibility, we join *m* **ip_t** (Table Name: $it_{1},it_{2},\cdots ,it_{m}$), *n* **op_t** (Table Name: $ot_{1},ot_{2},\cdots .,ot_{n}$) and m+n **tran_taxo_t** (Table Name: $tt_{1},tt_{2},\cdots ,tt_{m+n}$) with four kinds of conditions, *Selection conditions for the given m input events and n output events, Conditions for ancestor-descendant relationships, Join conditions for connecting input/output tables, Conditions for uniqueness.*

The complete SQL query for the IO query in the basic table design is shown in Figure 12b. The SQL query in the basic table design has m+n joins with **tran_taxo_t** and the size of *tran_taxo_t* can be big. Therefore, executing the SQL query in the basic table design may spend much time. In the advanced table design, we can avoid joins with **tran_taxo_t** using region numbers. To translate the IO query into the SQL query in the advanced table design, we should first obtain the region numbers of the given input/output events ($ip_{1},ip_{2},\cdots ip_{m},op_{1},op_{2},\cdots ,op_{n}$) from *taxo_ad_t*. If we let the region numbers of $ip_{i}$ (resp., $op_{i}$) be $ip_{i}\_start$ and $ip_{i}\_end$ (resp., $op_{i}\_start$ and $op_{i}\_end$), we add the conditions for the ancestor-descendant relationship between two events as follows:

$it_{1}.event\_start<=ip_{1}\_start\;and\;it_{1}.event\_end>=ip_{1}\_end$, ⋯,

$it_{m}.event\_start<=ip_{m}\_start\;and\;it_{m}.event\_end>=ip_{m}\_end$,

$ot_{1}.event\_start<=op_{1}\_start\;and\;ot_{1}.event\_end>=op_{1}\_end$, ⋯,

$ot_{n}.event\_start<=op_{n}\_start\;and\;ot_{n}.event\_end>=op_{n}\_end$

The complete query for the IO query in the advanced table design is shown in Figure 12c. The join conditions for connecting input/output tables and the conditions for uniqueness are added such as the IO query translation in the basic table design.

### 8.2. Structure Query Processing

Algorithm 1 shows a method to translate a structure query into the SQL query in the basic table design. Since a structure query consists multiple block statements, we translate each block statement into the partial SQL statement and merge them to make the complete SQL statement. In Line 1, we initialize sqlQuery which has the *SELECT* part, the *FROM* part and the *WHERE* part. In Lines 2–12, each block statement $BS_{i}$ is translated to the partial SQL query. It is appended to sqlQuery. We add *tran_block_t* $B_{i}$ to the FROM clause of sqlQuery (Line 3). $B_{i}$ can be considered to be the block table name corresponding to $BS_{i}$. To process the ancestor/descendant relationship between blocks, we must use *tran_block_t* instead of *block_t*. If the block statement has its parent block id (i.e., the block statement is not in the top), we add the join conditions with the parent block according to the relation types (Lines 5–9). If the parent block ($B_{p}$) and the current block ($B_{i}$) are connected indirectly (i.e., the ancestor/descendant relationship), we add the join condition for the relationship, *$B_{i}$.parent_block_id = $B_{p}$.block_id* (Lines 6–7). Additionally, to prevent blocks in different models from being joined, we add the condition, *$B_{i}$.model_id = $B_{p}$.model_id*. If the parent block ($B_{p}$) and the current block ($B_{i}$) are connected directly (i.e., the parent/child relationship), we add the join conditions similar to the indirect case above (Lines 8–9). However, in this case, we add the additional condition *$B_{i}$.iteration=1*.
**Algorithm 1:**translateStructureQuery_BASIC()
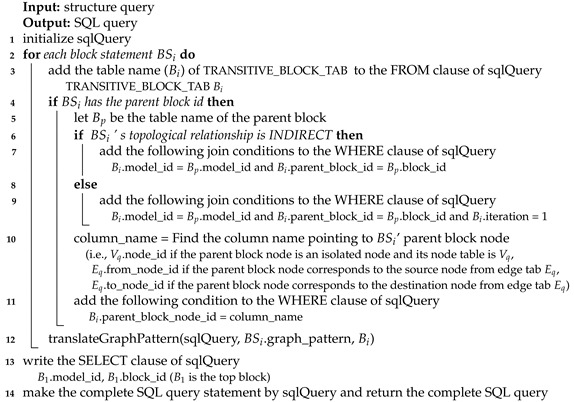


By the conditions above, we can connect the current block to the parent block. However, we should also add the condition for the parent block node because the current block is connected to some node in the parent block. To do that, we add the condition $B_{i}.parent\_block\_node\_id=column\_name$ (Lines 10–11). column_name is the column name pointing to the parent block node. In Line 12, we translate the graph pattern into the SQL query which will be described later. Finally, we can make the complete SQL query in Lines 13–14.

Algorithm 2 shows an algorithm to translate a structure query into the SQL query in the advanced table design. In Lines 2–15, each block statement is translated to the partial SQL query and in Lines 16–17, the final SQL query is made. In the advanced table design, we use *block_ad_t* instead of *tran_block_t* (Line 3). If the block statement has its parent block id (i.e., the block statement is not in the top), we add the join conditions with the parent block according to the relation types (Lines 4–14).

If the parent block ($B_{p}$) and the current block ($B_{i}$) are topologically on the ancestor/descendant relationship, we add the conditions in Line 8. The ancestor/descendant relationship between two blocks is identified using region numbers of *block_ad_t*. Even though the region numbers of *block_ad_t* can identify block relationships, they cannot check the parent block node. Because the child block can come from any node in the parent block, we should check parent_block_node_id. To give the condition on parent_block_node_id, we add auxiliary *block_ad_t*, which is a block node directly below $B_{p}$, to the FROM clause (Line 7). Additionally, we add the conditions to the WHERE clause as shown in Line 8. $B_{i\_aux}$ corresponds to a child block of $B_{p}$ and $B_{i}$ corresponds to a descendant block of $B_{p}$ including a child block. After that, we add the condition on $B_{i\_aux}.parent\_block\_node\_id=column\_name$ (Lines 9-10). If the parent block ($B_{p}$) and the current block ($B_{i}$) are topologically on the parent/child relationship, we add *$B_{i}$.model_id = $B_{p}$.model_id* and *$B_{i}$.parent_block_id = $B_{p}$.block_id* to give the join conditions for the parent-child relationship (Line 12) and we add the condition on parent_block_node_id (Lines 13-14). This part is almost the same as that of Figure 1. However, the part does not include $B_{i}.iteration=1$ because *block_ad_t* has only one record for the parent node while *tran_block_t* has many parent nodes (i.e., ancestor nodes) for each node.
**Algorithm 2:**translateStructureQuery_ADVANCED()
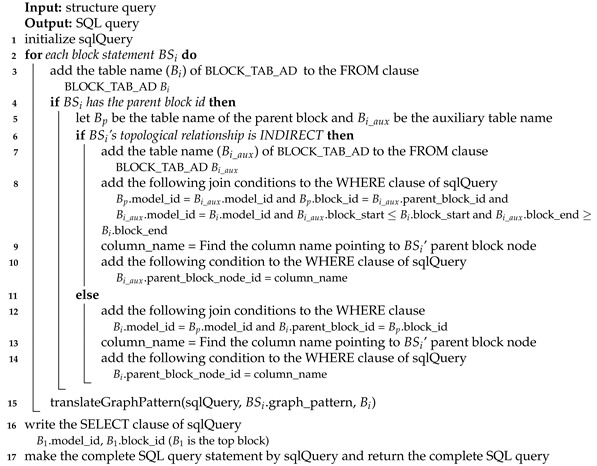


Algorithm 3 shows an algorithm to translate the graph pattern in both the basic table design and the advanced design (i.e., **translateGraphPattern() algorithm**). Basically, we map each edge to the edge table (i.e., *compact_dedge_t*) and we add the join conditions with the block to consider edges in only the same block. After that, we add conditions of connecting edges according to the node type. For brevity, we omit the detailed explanation of the algorithm.
**Algorithm 3:**translateGraphPattern()
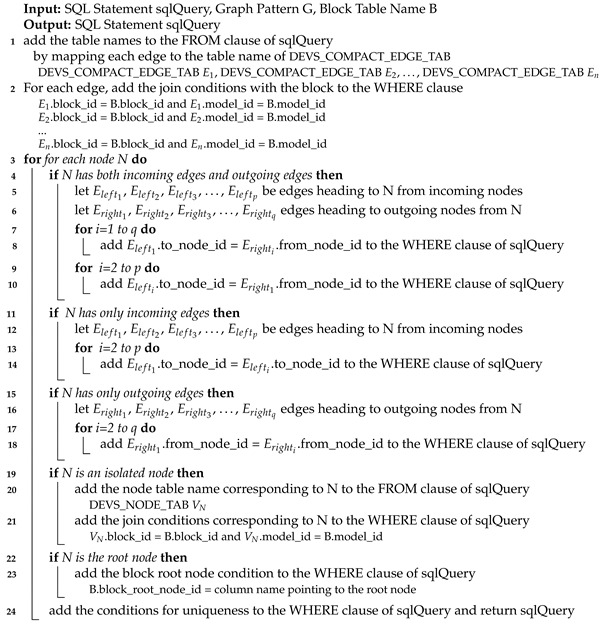


## 9. Experiments

In this section, we will show experimentally that GO-DEVS can process IO queries and structure queries efficiently.

### 9.1. Experimental Environment

To measure the performance of GO-DEVS, we generated synthetic data and queries. The reason we use synthetic data and queries is because this work is the first system to support storing and retrieving many DEVS models using ontologies to the best of our knowledge and therefore we could not find DEVS models whose input and output messages are designed using ontologies. We ran queries on Intel CPU (core i7-1.80 GHz) with 24.0 GB of memory and used MariaDB 10.5 as an RDBMS. The query execution time was measured by running queries 10 times and averaging them. To improve the query performance, we added indices in both the basic table design and the advanced table design properly.

GO-DEVS uses a taxonomy for DEVS sharing. To generate the taxonomy data, we used the well-known synthetic XML data [27,28]. Even though the synthetic XML data are not directly relevant to the taxonomy of GO-DEVS, the synthetic XML data show complex structures and hierarchies. Therefore, we are sure that it is appropriate for performance comparison. The generated XML data size is approximately 1MB and the number of nodes in the XML data are 17,132. Each XML node corresponds to an input or output event in DEVS models. The maximum depth of XML data are 11 and the average depth is 4.5.

We generated DEVS models by considering the number of subcomponents in a block, component hierarchies and relationships between subcomponents. By *<Hierarchy factor>*, we determine if we generate an atomic model or a coupled model. The range of *<Hierarchy factor>* is [0, 1). When the random number (∈[0,1)) is more than *<Hierarchy factor>*, we generate the atomic model and stop the generation process. Otherwise, we produce *m* subcomponents and their couplings, where *m* is the random number between 1 and *<Maximum components in a block>*. For each subcomponent, the process above is repeated. We set *<Hierarchy factor>* and *<Maximum components in a block>* to 0.3 and 5, respectively.

For input/output ports of an atomic model, we made the candidate input/output port (or event) sets in various sizes and chose one from the candidate input/output port sets to generate input/output ports of the atomic model. The number of ports in an atomic model ranged from *<Minimum number of ports in an atomic model>* to *<Maximum number of ports in an atomic model>*. *<Minimum number of ports in an atomic model>* and *<Maximum number of ports in an atomic model>* were set to 2 and 6, respectively. A candidate port set was produced by randomly picking up the event from event samples which were constructed by selecting some XML nodes between level 3 and level 10 (Root level 0). Additionally, to avoid IO queries with no results, we generated the IO-compatible port set with the above produced port set. For a coupled model, we generated *n* internal couplings where *n* was generated randomly between 0 and (the number of subcomponents in a block)*<Degree>/2. <Degree> was set to 2. For input and output ports that are not connected by internal couplings, we connected them to their main model by EIC and EOC, respectively. Finally, we generated 30,000 DEVS models based on the description above.

To evaluate the performance of GO-DEVS, we made 8 IO queries (Q1 to Q8) and 6 structure queries(Q9 to Q14). We varied the number of ports for various experiments of IO queries (Q1/Q2 = 1 input/output port, Q3/Q4 = 2 input/output ports, Q5/Q6 = 3 input/output ports, Q7/Q8 = 4 input/output ports). Input and output ports of Q1, Q3, Q5 and Q7 were chosen from event messages in low level on the taxonomy (i.e., depth = 3) and those of Q2, Q4, Q6 and Q8 from event messages in high level (i.e., depth = 6).

**Figure 13:**
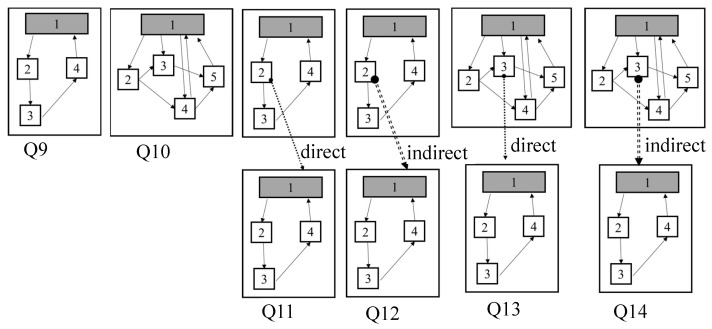
Graph Patterns of Structure Queries.

Structure queries are specified with block statements as shown in Figure 13. Figure 13 shows the graph patterns of structure queries. Q9 and Q10 are a simple structure query with only one block statement. Q10 has more nodes and edges than Q9. Q11 to Q14 have two block statements and block statements are connected. In Q11 and Q13, block statements are connected via the parent/child relationship while, in Q12 and Q14, block statements are connected via the ancestor/descendant relationship. Additionally, Q13 and Q14 have similar structures to Q11 and Q12, respectively. However, Q13 and Q14 have more nodes and edges in the first block compared to those of Q11 and Q12.

### 9.2. Experimental Results

**Figure 14:**
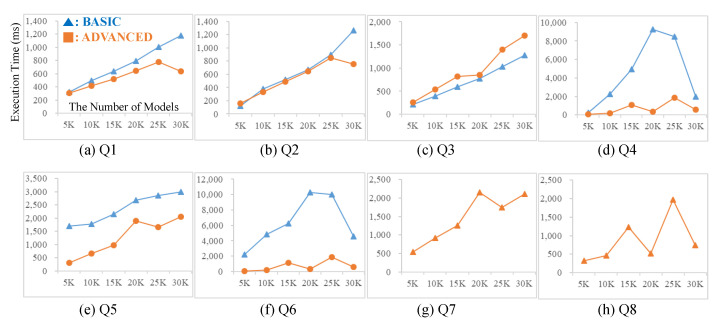
Experiments for IO Queries.

With the data sets and the queries described above, we measured the query performance of the basic table design and the advanced table design. To conduct experiments according to data size, we extracted six data sets in different sizes (i.e., 5 K, 10 K, 15 K, 20 K, 25 K and 30 K) from the generated 30,000 DEVS model data. Figure 14 shows the experimental results for 8 IO queries. In most cases, the execution times of the advanced table design were better than those of the basic table design. For experiments of Q7 and Q8 in Figure 14, we did not depict the execution times of the basic table design since the queries in the basic table design were not finished during long time (300,000 ms). The experimental results for IO queries showed a tendency that the performance gap between the advanced table design and the basic table design rises as the number of IO ports increases.

In addition, let us observe the execution times of Q3 and Q5 (low level ports) compared to those of Q4 and Q6 (high level ports), respectively. The performance gap in queries with high level ports was higher than that in queries with low level ports. In particular, in Q3, the advanced design was a little worse than the basic design while in Q4, the advanced design was much better than the basic design. This is because for queries with high level ports, the basic table design needs many ancestor event records in *tran_taxo_t* to check the relationship while the advanced table design needs only one record in *taxo_ad_t*.

**Figure 15:**
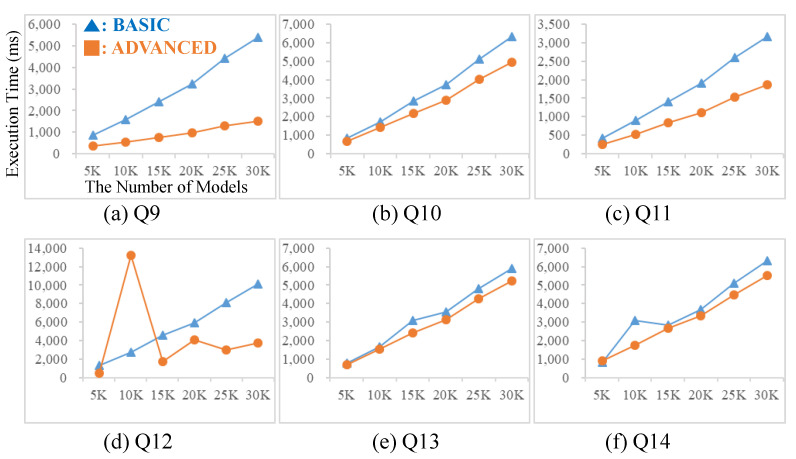
Experiments for Structure Queries.

Figure 15 shows the experimental results for structure queries. Q9 and Q10 have one block statement and Q9 has a simpler graph pattern than Q10. Therefore, the query result size of Q9 is bigger than that of Q10. In both Q9 and Q10, the advanced table design is better than the basic table design in terms of the query execution time. Q11 and Q12 have two block statements and they have the same graph patterns. However, two block statements are connected by the parent/child relationship in Q11 while they are connected by the ancestor/descendant relationship in Q12. Q13 (resp., Q14) has a similar structure to Q11 (resp., Q12) but Q13 (resp., Q14) has more nodes and edges in the first block than Q11 (resp., Q12). Now let us look at the experimental results of Q11, Q12, Q13 and Q14. The advanced table design showed a better performance compared to the basic table design in most cases. The query execution times of the advanced table design in Q12 fluctuated. This is because the SQL query optimizer did not work well for that query.

Consequently, we showed that our two approaches, *basic and advanced table designs*, can be used to process IO queries and structure queries in GO-DEVS. In particular, the advanced table design is much faster than the basic table design for IO queries that have many IO ports or have high level ports. For structure queries, the advanced table design can efficiently process queries compared to the basic table design in most cases. Therefore, model developers can retrieve some shared components effectively and efficiently using two types of queries.

## 10. Discussion

Even though we tried to develop a storage and retrieval system for simulation model sharing, limited to DEVS, our approach has some limitations and there are several untouched issues to be solved in the future. To promote the practical use of simulation model sharing, not limited to DEVS, researchers in both M&S (Modeling and Simulation) and DB (DataBase) areas should collaborate. In this paper, we provided an initial framework for model sharing in terms of DEVS. However, there are several untouched issues as follows:

- **DEVS extensions:** We developed GO-DEVS by focusing classic DEVSs but there are many DEVS extensions. To share as many models as possible, we should consider all the DEVS extensions in a model storage and retrieval system. To support them, we can consider a framework to provide both common table designs and user-defined table designs. We can extract common design concepts from a classic DEVS and various DEVS extensions. Based on the common design concepts, the classic DEVS and simple extensions can be handled. For other DEVS extensions, users must add a user-created design using tools provided by the framework.
- **Integration of SES and our approach:** As mentioned in Section 2, SES (System Entity Structure) was proposed for “plan-generate-evaluate” framework [2,13]. It can be used for representing the structural knowledge in hierarchical and modular systems. Even though SES can provide the structural information between parent and child components in a general way, it does not consider the relations between components at the same level. Therefore, SES and our approach need to be integrated in a systematic way. For instance, we can extend SES by adding internal structures and develop a framework to store and query extended SES data.
- **Query Extensions:** Even though we proposed IO and structure queries, we need to invent easy and powerful queries for model sharing. Based on the queries, various query optimization techniques must be developed to provide users with query results quickly. Additionally, we must develop a UI tool for users to input queries easily.

## 11. Conclusions

In this paper, we proposed GO-DEVS to systematically store and retrieve many DEVS models. We introduced the ontology concept to effectively share DEVS models developed by other model developers. To store DEVS models, we proposed a method to translate a DEVS model into graph representation. Additionally, we adopted an XML numbering scheme to process IO queries and structure queries efficiently and provided two relational table schemas, *basic table design* and *advanced table design*, to save DEVS models into an RDBMS. We finally showed that our two approaches can be used to process IO queries and structure queries and the advanced table design is better than the basic table design in most cases in terms of the execution time.
